# Biosensors Based on Mechanical and Electrical Detection Techniques

**DOI:** 10.3390/s20195605

**Published:** 2020-09-30

**Authors:** Thomas Chalklen, Qingshen Jing, Sohini Kar-Narayan

**Affiliations:** Department of Materials Science, University of Cambridge, Cambridge CB3 0FS, UK; tac49@cam.ac.uk

**Keywords:** biosensors, mechanical biosensor, electrical biosensor, MEMS biosensor

## Abstract

Biosensors are powerful analytical tools for biology and biomedicine, with applications ranging from drug discovery to medical diagnostics, food safety, and agricultural and environmental monitoring. Typically, biological recognition receptors, such as enzymes, antibodies, and nucleic acids, are immobilized on a surface, and used to interact with one or more specific analytes to produce a physical or chemical change, which can be captured and converted to an optical or electrical signal by a transducer. However, many existing biosensing methods rely on chemical, electrochemical and optical methods of identification and detection of specific targets, and are often: complex, expensive, time consuming, suffer from a lack of portability, or may require centralised testing by qualified personnel. Given the general dependence of most optical and electrochemical techniques on labelling molecules, this review will instead focus on mechanical and electrical detection techniques that can provide information on a broad range of species without the requirement of labelling. These techniques are often able to provide data in real time, with good temporal sensitivity. This review will cover the advances in the development of mechanical and electrical biosensors, highlighting the challenges and opportunities therein.

## 1. Introduction

Biosensors can be broadly defined as devices that are used to detect the presence or concentration of a biological analyte [1,2,3,4,5,6]. This may take the form of a biomolecule, a biological structure or even larger structures such as cells and microorganisms. Biosensors typically consist of four fundamental parts: the analyte under examination, the binding substrate to which the analyte attaches, a transducer to produce a recognisable signal from the binding event, and a data processor to convert that signal to a meaningful value.

The first biosensor, created by Clark and Lyons in 1962, was fabricated to detect glucose using a glucose oxidase enzyme to convert glucose into gluconic acid. The gluconic acid lowered the pH of the solution in proportion to the glucose concentration, enabling the detection of glucose levels in samples [1]. This was a significant milestone for medicine as, for the first time, it was possible to monitor blood glucose, rather than relying on glucose concentrations in urine samples. This eventually set the stage for present-day electrode glucose sensors, which allow for instantaneous measurement of blood glucose levels [7,8]. The motivation behind the development of glucose biosensors is obvious; they represent a large global market, given that there are currently 422 million cases of diabetes globally and diabetes is the cause of 1.6 million deaths annually, according to the World Health Organization (WHO) [9,10]. At present, biosensors can be used to study a wide range of analytes, ranging from small molecules (such as glucose) through DNA, antigens and antibodies, to whole cells and even full tissue monitoring in some cases [6,11,12,13] Depending on the specific analyte in question, biosensors can be designed to detect a range of signals, but are most commonly used to determine the concentration of a given species. They often rely on the target molecule itself being able to provide information about a specific disease or condition in question, for example, in the case of glucose using its concentration to diagnose and monitor diabetes [14,15,16].

**Figure 1:**
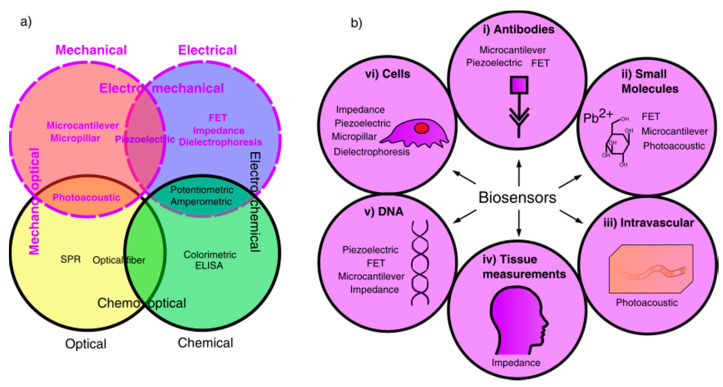
Schematic diagram representing biosensor applications and fields. (**a**) Biosensors categorised into different fields based on detection and signal transduction methods; mechanical and electrical fields are highlighted as the area of study for this review. (**b**) Biosensor applications of chosen fields, showing suitable biosensors for detection of different analytes, including: (i) antibody detection, (ii) small molecule detection, (iii) intravascular detection, (iv) full body measurements, (v) DNA detection, (vi) cell measurements.

Historically, the majority of biosensor research has been primarily focused on chemical, electrochemical and optical methods of detecting an analyte. Therefore, this review will instead focus on mechanical, electrical and electromechanical biosensors, as summarised in Figure 1, which shows the fields of available biosensors, highlighting those using mechanical or electrical detection methods, and the applications of mechanical and electrical biosensors covered in this review. 

In order to appreciate the biosensors covered here it is useful to examine some of the more frequently occurring biosensors currently in use. This includes the ubiquitous lateral flow tests most commonly found in pregnancy tests, and also the commonly used enzyme linked immunosorbent assays (ELISA). Both of these tests produce a colour change in the fluid tested, either using bound fluorescent tags or a bound enzyme [17,18] They can be very effective for the detection of a particular analyte, for example, being able to detect human immunodeficiency virus (HIV)-1 capsid antigen p24 down to concentrations of 10^−18^ g mL^−1^ [19]. Whilst hugely effective and straightforward to operate, there are severe limitations to these methods, namely, the specificity of the assays limits their usefulness for more general diagnostic methods, and the assay can only provide information on the presence or absence of a particular analyte, but not the concentration of the analyte. 

Other optical-based methods can be significantly more complicated to carry out and interpret. For example, a large number of optical biosensors involve the use of surface plasmon resonance (SPR) to track the adsorption of analyte. Whilst highly sensitive, the sensitivity is affected by a number of factors, and is particularly dependent upon surface functionalization [20] which can be difficult to achieve [21]. The interpretation of the results is also challenging, as SPR is affected by the entire enzyme substrate complex, meaning that, without an understanding of the reaction mechanism, results are of limited use [22]. Furthermore, in order to function, the incoming light must be polarised to match that of the plasmon resonance, which adds complexity to the setup. This method is also quite slow, taking roughly 20 min for a single measurement and several hours for multicycle measurements [23,24,25].

An issue for most biosensors, including SPR-based biosensors, is that they rely on some prior knowledge of a particular species to search for. If a species is unknown, say for example, running a blood test for an unknown pathogen, these sensors cannot provide any useful information. These sensors are then effectively operating on a best-guess trial-and-error basis. There is, therefore, a need for biosensors that can provide rapid quantitative information on unknown analytes. There is also a requirement for real-time measurements, and, given the complexity of many existing techniques, a need for relatively straightforward techniques to be accessible to people without a specific background in the relevant technology, such as in home-based testing kits. Given the general dependence of most optical and electrochemical techniques on labelling molecules, this review will focus instead on electrical and mechanical techniques, as they can provide information on a broad range of species without the requirement of labelling. In addition, electrical and mechanical techniques are often able to provide data in real time, with good temporal sensitivity. In the future, this may enable the assessment of biomolecules on single cells in real time. This could have the potential to revolutionise a number of fields ranging from neuroscience to cell biology. As an example, consider that the ability to monitor a single cell during its lifespan could help to shed light on the causes of Alzheimer’s disease, which remains a major unsolved challenge [26,27]. This review will cover the advances in the development of mechanical and electrical biosensors, highlighting the challenges and opportunities therein.

## 2. Mechanical Biosensors

### 2.1. Microcantilevers

Microcantilevers (MCs) have emerged as an important field of study for highly sensitive biosensors. They function by the attachment of the analyte in question onto the surface of a microscale cantilever, usually made of silicon. There are two different modes of operating the cantilever; a static mode, in which the analyte attachment generates a surface stress, causing deflection of the MC, and a dynamic mode, where the binding of the target molecule alters the resonant frequency of the MC. There are a number of differences between the design of static or dynamic MCs. For the dynamic mode, a short and stiff cantilever is desirable to give higher resonant frequencies, as high frequencies are less sensitive to low frequency background noise. On the other hand, for the static mode, it is preferable to have a long flexible cantilever to maximise the deflection [28]. Dynamic mode MCs also require some form of actuation to reach their resonant frequency [28,29]. This makes the desired materials and design quite dissimilar, hence they will be considered separately here.

In general, MCs exhibit unprecedented sensitivity, frequently being used to detect compounds at concentrations below 50 fg mL^−1^, and are therefore developing into an important field [30,31]. However, MCs suffer from a number of issues. In the static mode, it is challenging to only attach receptors to one surface [32], while in the dynamic mode, the shift in resonant frequency is not just dependent on the mass of the target molecule, but also its effect on flexural rigidity, which acts contrary to the mass detection mechanism [33]. For both modes, the signal resulting from a given particle will also vary depending on the binding location and surface energy, which may be influenced by the surrounding medium [30]. Sensitivities are therefore a lot higher in vacuum than air or fluid. Nevertheless, the detection limits approached by this technology are steadily going beyond that achievable by SPR, which is considered to be the gold standard in biosensing [34].

#### 2.1.1. Static Mode MCs

**Figure 2:**
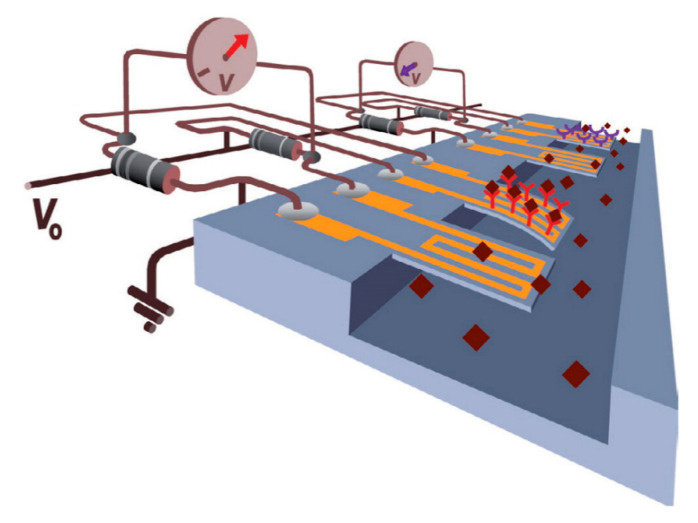
Schematic of the cantilever-based measurement system proposed by Boisen and Thundat. The bending of the cantilever caused by the attachment of the molecules changes the resistance of the built-in resistor. Two adjacent units, one of which serves as reference, are measured simultaneously and only the difference between signals is recorded. Reprinted with permission from [35]. Copyright © 2009 Elsevier Ltd.

Static MCs, by virtue of their low thickness (and hence distance from the cantilever neutral axis), are highly flexible, with a low second moment of area. This gives a greater deflection for small stresses, and hence signal, for low analyte concentrations than is achievable via other methods. A schematic illustration of a static microcantilever is shown in Figure 2. There are a number of challenges associated with this technique. Firstly, in order to cause deflection, the analyte must only attach to one surface. Preventing functionalisation of the bottom surface whilst functionalising the top is complex [32]. Secondly, the detection and calibration of small deflections can be problematic. A few different methods for monitoring deflection have been developed to address this issue. The most common relies on piezoresistivity, and usually uses a silicon semiconductor cantilever [28]. As the beam bends the strain changes the interatomic spacing, resulting in a change in electrical resistivity. This change is usually measured differentially, by comparison to a reference microcantilever that is non-functionalised, in a Wheatstone bridge configuration [35], as shown in Figure 2.

**Figure 3:**
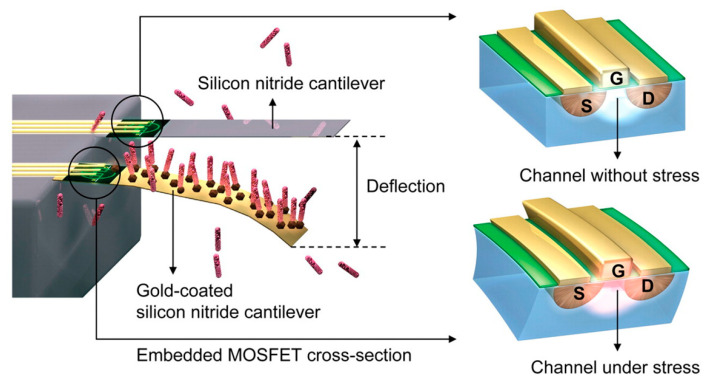
Schematic of the attachment and bending caused by target molecules on the probe on an embedded-metal–oxide–semiconductor field-effect transistor (MOSFET), proposed by Shekhawat. The silicon nitride cantilever acts as reference. The drain current of the MOSFET will change under local stress due to the conductivity modulation of the channel underneath the gate, hence the targeted molecules being sensed. Reprinted with permission from [36]. Copyright © 2006, American Association for the Advancement of Science.

A more effective approach involves using the piezoresistive effect to change the signal from an integrated field-effect transistor (FET), whereby the strain in the cantilever reduces the mobility of electrons in the base, thereby reducing the drain current [36]. This change can be calibrated to give the deflection of the cantilever and hence the adsorption of the analyte, as shown in schematically in Figure 3. Another method is to rely on a laser beam reflecting off the cantilever, similar to the optical detection mechanism used in atomic force microscopy (AFM) techniques [37]. However, this has its drawbacks as an array of sensors would require an array of lasers and detectors, which is both complex and expensive [32]. Furthermore, the refractive index of the surrounding fluid, fluid flow over the cantilever and heating effects from the laser can introduce difficulties in accurately measuring cantilever deflection.

In recent years, improvements to static MCs have involved the integration of microfluidics with MC arrays to improve the functionality of the device [38,39]. One advantage of this approach is that it may be used with re-usable MC surfaces, creating a re-usable device that can be flushed to regenerate the surface after use [40,41,42]. This has attracted a great deal of attention in recent years because of the capability to reduce the costs of MC biosensors, and improve repeatability of measurements. Another benefit to the combination of MCs and microfluidics is the capacity to use an array of multiple cantilevers in the same fluid. Either to measure the concentrations of multiple difference analytes or to improve the reliability of measurement with a single analyte [35,38,43,44].

#### 2.1.2. Dynamic Mode MCs

Dynamic mode MCs rely on binding events that change the resonant frequency of the cantilever. Monitoring this change can be used to quantify the degree of adsorption and hence the analyte concentration. The detection of the change in resonant frequency is achieved in a similar manner to static mode MCs, using piezoresistors, FETs or optical detection methods. However, in addition to detection, dynamic mode MCs also require actuation to function [28,29]. This actuation can be generated by electrostatic forces, piezoelectric elements or optical devices, with the frequency measurement also using the same approach [29]. Optical-based measurements of resonant frequency are the most widely used, either using an optical lever (as with AFM techniques) [45] or with a laser doppler vibrometer [46].

Resonating cantilevers can demonstrate very impressive sensitivity, with detection limits in the fg mL^−1^ range [30,47]. This sensitivity does come at a cost however, as the cantilevers cannot function well in a fluid medium due to large damping effects [30]. Hence these samples must be dried and measured in vacuum, which increases the length of preparation, reduces the relevance of the data, and prevents real-time measurements. Fortunately a solution to this issue has been presented by Burg et al. who created a suspended microchannel resonator [48]. The resonator has a very small volume of fluid running through it (~30 pL). As the analyte moves through, it binds to the inside wall of the microchannel, thereby increasing the mass and changing the resonant frequency. Thus, the resonator is able to detect picogram quantities of analyte in a fluid environment [48,49]. Such suspended microchannel resonators eliminate the requirements for testing to be carried out under vacuum, and the use of microfluidics greatly reduces sample preparation time and complexity. It has been shown that, by using multiple measurements with different fluid densities, it is possible to determine not only the mass of a cell but also its volume [50,51] These devices have been shown to have very high sensitivity despite being operated in fluid, with a detection limit of 0.12 pg for bacteria reported by Calmo et al. [52], and separately by Lee et al. in detecting single gold nanoparticles weighing 80 ag each [53].

**Figure 4:**
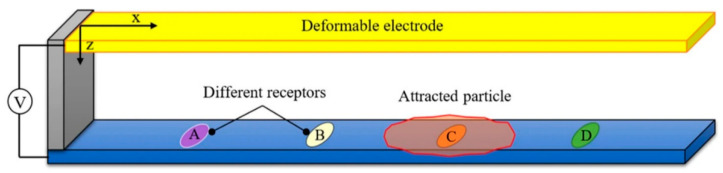
A schematic of cantilever type biosensor that can detect different biological components. Spots A-D indicate different receptors while particles were assumed to be attracted at spot C, as an example. Reprinted from [54] under CC BY 4.0. (https://creativecommons.org/licenses/by/4.0/).

Nevertheless, there are issues with this type of sensors. As sample volume decreases, the possible throughput also decreases. In the specific example from Lee et al. [53], it would take 11 days to process 1 µL of sample, and so despite extraordinary sensitivity, the practical uses may be limited. Another issue for MCs is that they require functionalisation before they can measure an analyte. However, more recently, SoltanRezaee et al. have proposed a different type of MC sensor that may be used to test for multiple biomolecules simultaneously [54]. It utilises electrostatic attraction between two parallel plates with an applied potential difference. By testing the pull-in voltage required to cause instability in the beam deflection, the analyte in question may be identified, by the reduction in available surface for electrostatic attraction. A schematic illustrating this principle is displayed in Figure 4. Though yet to be experimentally verified, this presents an exciting opportunity to test multiple compounds simultaneously by immobilising them on different sections of the substrate, which greatly increases the versatility of this test versus other single test MCs.

One of the major issues with all MC devices is that, at low concentrations, binding events to the cantilever may not occur uniformly across the device. If this is the case then the signal induced by a binding event near the base of the cantilever will give rise to a signal of a smaller magnitude than that induced by binding near the free end of the cantilever [33]. Therefore, a uniform cantilever is not necessarily the optimum geometry and other designs, such as that of a “trampoline”, should be considered [30,33]. This work also highlights an issue for dynamic mode MCs, which is that the increase in thickness of the effective cantilever (from target binding) increases its flexural rigidity, which will increase the resonant frequency of the cantilever. This is in opposition to the decrease in resonant frequency that is associated with the additional mass of the target [33], which makes deconvoluting the effects of analyte binding on resonant frequency a challenge for each different analyte.

### 2.2. Photoacoustics

**Figure 5:**
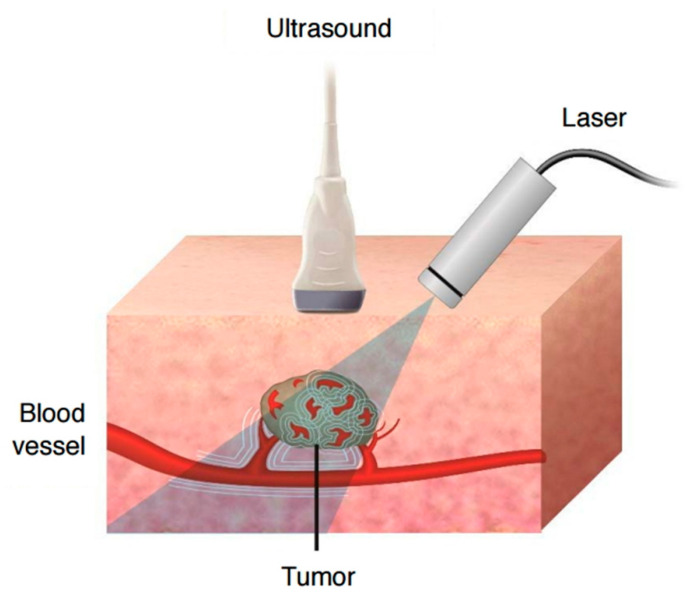
Schematic mechanism of photoacoustic effect and imaging. Tissue constituents absorb pulsed near infrared laser and undergo thermoelastic expansions, which generate ultrasound signals that can be then detected by ultrasound detector. Reprinted from [60] under CC BY 4.0. (https://creativecommons.org/licenses/by/4.0/).

Photoacoustic (PA) imaging functions through the use of a laser to generate ultrasound waves via the thermoelastic effect. It is effective for providing a complete non-invasive image of tissue, including blood vessels, down to depths of several centimetres, and providing resolutions of a few micrometers [55]. A schematic of PA imaging of tissue is shown in Figure 5. In addition to imaging tissue, PA techniques can detect molecules of interest, which is exciting as it presents the opportunity for non-invasive glucose monitoring, as an example [56]. It has further been demonstrated to detect gases at concentrations of parts per trillion in air [57], as well as reactive oxygen and nitrogen species down to tens of micromolar concentrations at a depth of 1 cm using labelling molecules [58]. The drawbacks to PA imaging is the large, complex equipment and the fast photobleaching of the lasers [58,59]. Some of these issues may be solved with the use of LEDs instead of lasers, and the use of new improved transducers is reducing the complexity of the equipment.

Photoacoustics is a rapidly developing field, with multiple applications, from structural flaw detection to novel micropumps [61,62]. One of the primary uses of PA is in biological imaging, due to its ability to provide real-time imaging of the body enabling easy identification of tumours [63,64], as well as probing of reactive species in order to detect inflammation [58]. The technique relies on ultrasound emission from the thermoelastic effect. In brief, a laser is focused upon the analyte (usually biological tissue) under examination. Pulses of the laser cause localised heating which creates strain. Due to the rapid pulsing of the laser, this strain generation can be of very high frequency, producing ultrasound in the megahertz region. The magnitude of the ultrasound varies according to the analyte under examination and can enable a picture to be built up of the specimen under examination. An advantage of PA imaging over conventional optical microscopy, or even optical coherence tomography [65] is that it has a much higher penetration depth, being functional up to several centimetres deep, whilst still maintaining high resolutions [66]. Ultrasound is far better at penetrating through tissue and fluid than light [67]. Also, due to the high frequencies of ultrasound waves used, the wavelength of the ultrasound can be kept low, enabling higher resolution (as the resolution is limited by the wavelength akin to the Rayleigh diffraction limit in microscopes) [67].

There has also been interest recently in using PA technology in flow cytometry [68]. PA flow cytometry may prove superior to traditional fluorescent based flow cytometry due to the lack of complex sample preparation, as PA imaging does not require labelling antibodies [69]. A recent example from Gnyawali et al. exhibited comparable performance to fluorescent flow cytometry, but label-free in this case, for identification of red and white blood cell populations [70]. Another study by Cai et al. has shown that PA imaging can, non-invasively, detect malaria-infected red blood cells at a concentration of 1 in 10^9^ [71]. This is about 1000 times better than existing tests and can be measured in less than 30 min, or instantaneously with a decreased sensitivity. This demonstrates the potential for PA technology to revolutionise diagnostic procedures.

Whilst not requiring labelling to operate, PA can enable detection of certain species using labelling molecules called exogenous contrast agents [67]. However, the preparation and introduction of the labelling molecules can be complicated, and, as with any laser-based technique this method is susceptible to photo bleaching. Fortunately, Hariri et al. have demonstrated an alternative to lasers with LED technology instead. Due to its stable, low intensity light, photobleaching is greatly reduced, with the further advantages that the LED technology is smaller, simpler and cheaper, suggesting this will be an important area of research in the future [58].

**Figure 6:**
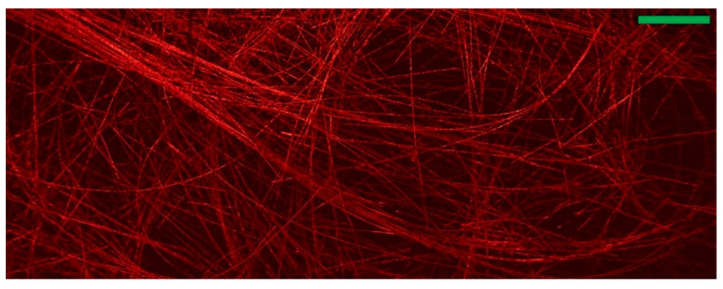
A mechanical scanning image showing carbon-fibre network. (Scale bar: 500 μm) Reprinted from [55] under CC BY 4.0. (https://creativecommons.org/licenses/by/4.0/).

Other recent improvements to this technology include the work of Hajireza et al. who demonstrated the imaging of 7 µm carbon nanotube networks as shown in Figure 6, by using two lasers; a pulsed laser excitation, with another, non-interfering beam [55]. The interaction of the pulsed beam with a refractive index boundary transiently amplified the difference in refractive indices, enabling effective imaging of material boundaries. The lack of coherence requirement between the beams also prevents the need for an optical medium, as is otherwise needed for ultrasound-based techniques. This method also demonstrated an incredible ~ 2.7 ± 0.5 μm lateral resolution. In a similar vein, recent developments by Zhang et al. have used a dual laser device to create a non-contact acoustic imaging set up that has been termed “laser ultrasound”. In this case, the pulsed beam was used to generate ultrasound at the tissue surface, in order to improve the penetration depth. The advantages of this method are a high penetration, down to depths of 5 cm, and a reduction in noise due to change in contact of the equipment and sample [72].

Photoacoustic imaging usually relies on an ultrasound transducer to convert the ultrasound waves into an electrical signal, however these tend to be large and have a low resonant frequency making them more susceptible to noise [73,74]. There has been a focus recently to improve the transducing mechanism for PA imaging, involving the use of piezoelectric transducers [60], quartz based transducers [75,76], and MC-based transducers [57,74]. These have found use as ultrasensitive gas detectors [57,77] because of their high sensitivity (in the parts per trillion range). As the transducer is one of the limiting factors in the effectiveness of PA imaging, and with the development of high sensitivity microelectromechanical systems (MEMS) as transducers, it is likely that transducer improvements will continue to be an area of interest for the foreseeable future.

### 2.3. Micropillar Sensors

Micropillar (MP) sensors are sensitive to mechanical deformation both transiently and continuously [78,79], and are relatively simple to fabricate (using silicon lithography techniques) [80,81] and to operate [80]. They function in a similar manner to MCs, but without the functionalised surface. Generally, the deflection of the pillar creates a stress on a piezoresistive material, changing the impedance of the circuit [79,82]. They are often used as arrays to detect forces exerted by cells through the deformation of the pillars [80,81]. This technique has great potential, though the current method of measurement using digital image correlation is slow and cumbersome [83]. In the future, it is expected that electrical measurements may improve the process. MP sensors may also prove relevant for biomedical sensors to measure fluid flow either intravenously or outside the body, for example with IV tubes [84] or blood transfusions.

**Figure 7:**
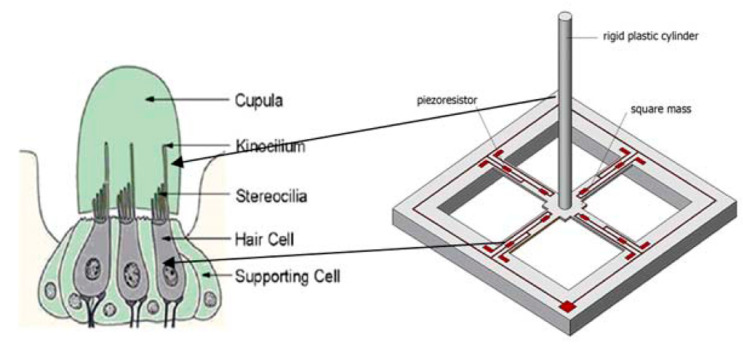
Schematic view of the MEMS hydrophone with bionic structure, proposed by Zhang. Reprinted from [90] under CC BY 4.0. (https://creativecommons.org/licenses/by/4.0/).

In recent years there has been significant interest in a variety of sensors that occur throughout the animal kingdom based on hairs [85,86,87,88,89,90]. This interest appears to stem from the effective mechanical detection such hairs offer. For example, the lateral line system is an array of hundreds of “neuromasts”—small hair cells—that can detect water flow and vibrations enabling navigation and movement in coordinated shoals by fish [91,92]. Neuromasts utilise the high aspect ratio of the hair, which makes them sensitive to vibrations, generating signals in the highly densely packed nerve cells that sit at the base of the hair. Successful replication of these mechanical sensors could have a range of technical and biological applications, including as underwater hydrophones to listen to underwater sounds, as shown in Figure 7.

As a result, artificial MEMS systems replicating hairs with MP sensors have been explored since 1987 for use in robotics [93]. More recently they have been utilised for underwater sensors to detect fluid flows in an analogous manner to the lateral line system of fish [94]. These sensors generally work using four underlying force sensitive resistors whose resistance changes in response to an applied force [94,95]. The direction of the applied force on the MP sensor may be determined spatially by correlating the change in resistance of each resistor with the orientation of the MP [79]. Alternative arrangements can use the same geometry as a microcantilever [96]. Optimisation of the geometry of hair sensors by Engel et al. have shown that the important geometric parameters are the diameter of the hair and the size of force-sensitive resistor, with a large pillar diameter and small resistors giving the greatest sensitivity [95]. They also demonstrated the feasibility of using an all-polymer sensor, which may be crucial for future implantable biosensors based on this device, due to the biocompatibility of most polymers [97,98]. Asadnia et al. created an array of silicon MPs, only 350 µm in diameter, mounted on top of PZT transducers and embedded in polydimethylsiloxane (PDMS) [99]. This enabled determination of the origin of disturbance in the fluid, with a threshold detection velocity of 8.2 µm s^−1^ in water. Another PDMS-based hair sensor has shown good sensitivity in gaseous environments, detecting pressure variations of 1 Pa in a gas flow [100]. Other interests in these sensors include replicating the function of actual hair cells in the ear. Lenk et al. have tested one such sensor, displaying high sensitivity, detecting sounds below 15 dB (quieter than a whisper) [78], though this has yet to be fully implemented. It has been suggested that the extraordinary sensitivity shown could be useful in the next generation of implantable biomedical sensors for example in monitoring heart valve function for backflow [82,84,101].

**Figure 8:**
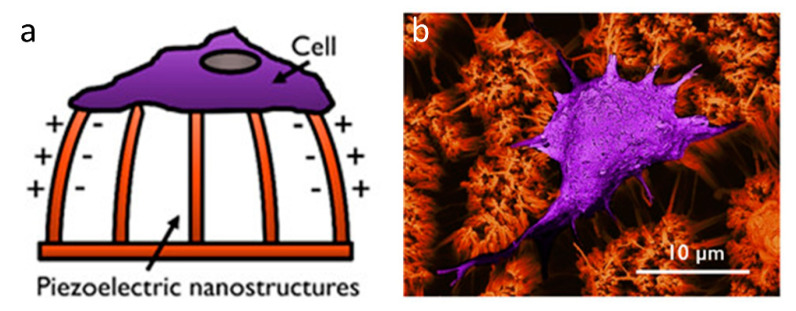
Piezoelectric cell force sensor. (**a**) Schematic of a cell force sensing device based on Poly-l-lactic acid (PLLA) piezoelectric nanostructures. (**b**) SEM image showing cellular interaction with PLLA nanotubes. Reprinted with permission from [107] (https://pubs.acs.org/doi/10.1021/acsabm.0c00012), Copyright © 2020 American Chemical Society (further permissions related to the material excerpted should be directed to the ACS).

A different use of MP sensors is to measure cell adhesion forces. At present this is one of the most important methods for measuring cell forces, as it is possible to track the forces applied by a single cell in tens or hundreds of places [80,102]. These forces are measured by tracking the MP deflection using optical methods [103,104], and then correlating the deflection to a force theoretically. This method has some issues however, as short pillars can be inaccurate by a factor of 40% due to deformation of the underlying base, which is not usually accounted for [105]. Another issue for MPs is quite fundamental, that the behaviours of cells on a 2D MP array is different from that on a flat surface or in an in vivo environment. It might be expected that in the future, densely packed arrays of micropillars might better represent native tissue. However they are currently limited in the range of stiffnesses currently in use [106]. Work in this area has already begun, as shown in Figure 8, but has yet to be fully integrated to measure cell forces [107]. Given the sensitivity of piezoresistive MPs, as well as the real-time response and the reduction in required computation from an electrical measurement it might be expected that future MP biosensors will use piezoresistive material instead of optical measurements. In either case it is likely that micropillars will remain an important tool for cell force measurement.

### 2.4. Piezoelectric Sensors

Piezoelectrics are an important avenue for biosensors, due to their sensitivity, affordability and relative simplicity, with generally simple fabrication techniques [108,109]. The majority of these function through the use of acoustic waves generated in a piezoelectric crystal [109]. As an analyte attaches to the surface, the frequency of the acoustic waves will decrease in response to the additional mass, in accordance with the Sauerbrey equation [110]. These acoustic wave sensors may be used for the detection of heavy metal ions such as Pb2+ [111], proteins [112], complex biological molecules [113]. The drawbacks of acoustic wave sensors are that, to increase their sensitivity the mass of the sensor itself must be reduced by decreasing the thickness. This ultimately limits the sensitivity due to the trade-off between mechanical stability and sensitivity, although, with the advent of flexible piezoelectric sensors, this limit may be improved [114]. However the main issue for acoustic wave biosensors is ensuring tight binding between the analyte and surface, as a weak binding will disrupt the measurement. Therefore, it is the issue of functionalisation of the surface that is still to be fully solved [112].

Piezoelectric biosensors have a long history, dating back to the discovery by Sauerbrey in 1959 that quartz crystal resonators show a linear relationship between deposited mass and frequency response of the crystals standing wave [115]. This led to the development of the first acoustic wave resonators, using thickness shear modes, which are commonly referred to as quartz crystal microbalances (QCM). They displayed very high sensitivity, of the order of ~1 ng. This was roughly 100 times better than the electronic balances of the time [116,117]. However it was not until 1982 when Nomura and Okuhara managed to successfully measure quartz crystal frequencies in fluid [118], that the stage was set for the use of QCM in biosensor applications.

**Figure 9:**
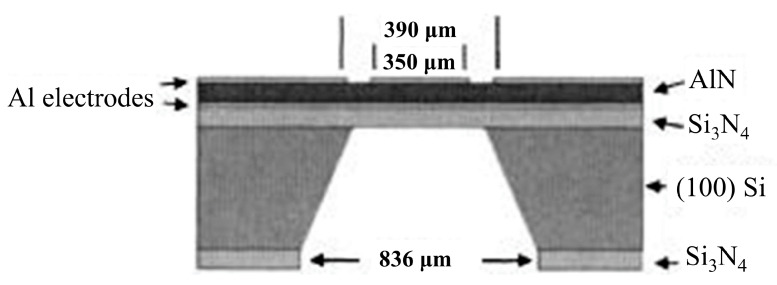
A cross-section structure of thin-film bulk acoustic wave piezoelectric resonator for microbalance chemical sensing. Reprinted with permission from [119]. Copyright © 2005 American Institute of Physics.

QCMs function based on the change in frequency of a piezoelectric crystal (quartz) in response to the adherence of a target molecule [112]. In order to increase the sensitivity, high-frequency small-area resonators, such as the one displayed in Figure 9, are used [119]. One limitation of this technique is that to function effectively, the analyte in question has to be strongly bound to the crystal, otherwise it will not produce a frequency shift [112,120]. This requires careful preparation in order to successfully adhere the analyte onto the QCM [113]. Preparation of these surfaces often requires the formation of a gold-sulphur bridge to enable the analyte to be strongly bound, enabling, for example, detection of DNA target concentrations greater than 50 ng mL^−1^ [121]. Another issue is that many other factors can affect the frequency, including temperature, pressure, conductivity and viscosity of the media [118,122]. Despite these limitations Kim et al. reported the use of an indirect-competitive QCM immunosensor to obtain a detection limit of 0.13 ng mL^−1^ [123]. A system proposed by Eidi et al. may help to improve repeatability and the detection limit of acoustic wave resonators, by having the sensing surface and the electrical connects on opposite surfaces to eliminate effects of media conductivity, although the actual measurement of such a system is yet to be reliably tested [124].

There are four different types of acoustic wave resonators depending upon the mode of vibration; thickness shear-mode resonator (e.g., the commonly used QCM), flexural plate-wave resonator, surface acoustic wave resonator and shear-horizontal acoustic plate wave resonator. In order to increase the detection limit and sensitivity of these devices, in recent years, attention has turned to the use of surface acoustic wave (SAW) resonators. This improves the frequency of operation from around 30–40 MHz for QCM, to 1 GHz for SAW devices [116,119], which correspondingly increases the detection limit and sensitivity, enabling detection down to a few parts per million (ppm). The advantage that these devices can provide is that they enable real-time detection over a long period, as reported by Jandas et al. who demonstrated a stable SAW sensor integrated with microfluidics to enable sensor regeneration, with a high sensitivity of 0.31 ng mL^−1^ and high stability over a 30 day period [108]. They used this device to monitor carcinogenembryonic antigen, a sign of a variety of different cancers, which implies the possibility of future use of such a device to monitor tumour development in real time, providing better treatment options. One of the limitations of many existing biosensors, particularly that of SPR-based biosensors (which are the main competition to QCM type biosensors) [112] is the ability to bind the analyte to the surface in question. This has for many years been limited by the suitability of different surfaces, requiring one that remains inert in a complex solution. Gold has been the most common choice as a result [108,109,121,125]. However more recently, with the more widespread understanding and availability of graphene, this is starting to be used as a detection surface. An example of this comes from the work of Ji et al. producing a SAW biosensor for the detection of bacterial endotoxin with a high sensitivity of 3.5 ug L^−1^ [126]. An alternative is the design developed by Lamanna et al. using a thin molybdenum layer on AlN instead of gold on quartz [114,127]. The advantage here is that the thin AlN layer is better able to accommodate strain, and hence the device is made flexible. This overcomes an inherent problem for SAW devices, whereby higher sensitivity requires thinner devices, which can then lose mechanical stability.

**Figure 10:**
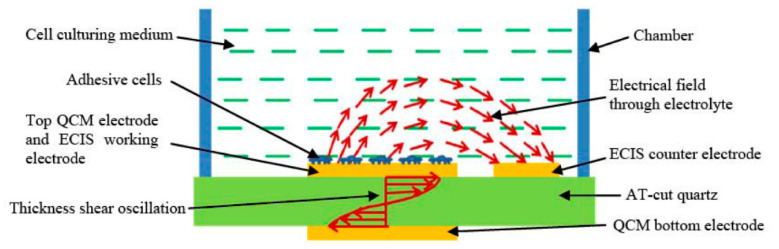
Illustration of the working principle of the hybrid biosensor developed by Liu. that combined QCM with ECIS. Reprinted from [125] under CC BY-NC-SA 3.0 (https://creativecommons.org/licenses/by-nc-sa/3.0/).

Another important development both for the QCM field and beyond is the integration of multiple sensors onto a single platform, as this provides significantly more information about the analyte than a single sensor can. This has been effectively realised by Liu et al. [125], who combined QCM with electric cell-substrate impedance sensing (ECIS), as illustrated in Figure 10. This produced a sensor capable of detecting both electrically and mechanically the development of < 500 bovine aortic endothelial cells during attachment, spreading and formation of a monolayer. They have further developed this to be used as a toxicity sensor with live cells, using the impedance change and frequency change of the ECIS and QCM as a proxy to measure the toxicity of various species [128].

Separately, in recent years, piezoelectric materials have become a focus for monitoring of vital signs. This is a potentially lucrative area, given the popularity of smart watches and the increased use of technology in professional sport [129,130], an area where considerable interest has been generated. For example, Allataifeh et al. have developed and tested a method using a lead zirconium titanate sensor, to deconvolute the strain caused by breathing from the vibrations of the heartbeat, allowing simultaneous non-invasive monitoring of vital signs [131].

### 2.5. Other Mechanical Biosensors

**Figure 11:**
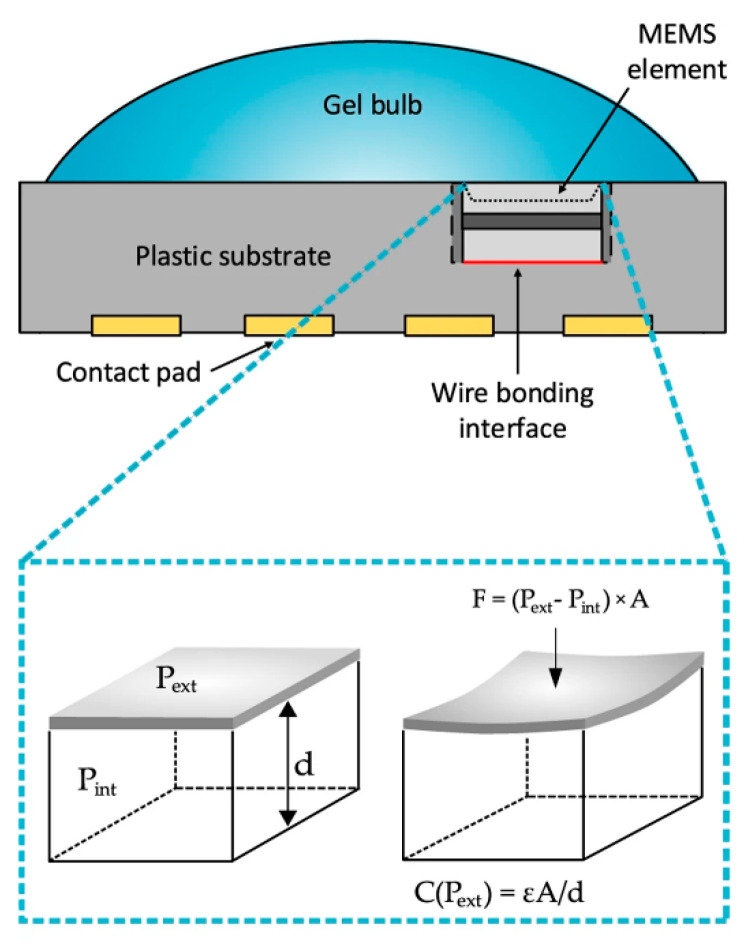
Schematic structure of the heart rate sensor that contains an MEMS pressure sensing elements. The capacitance of the elements changes according to the pressure change caused by the deformation of the diaphragm. Reprinted from [133] under CC BY 4.0 (https://creativecommons.org/licenses/by/4.0/).

Independently, two groups simultaneously used commercially available MEMS-based pressure sensors in conjunction with their own algorithms to measure heart rate [132,133]. Whilst one sensor is labelled as a pressure sensor, the other is referred to as a microphone, but they both function using a flexible membrane as part of a capacitor, as illustrated in Figure 11. As the membrane vibrates, the capacitance changes, which may be monitored by relevant circuitry. This provides an alternative to the more common photoplethysmography, which is found in the majority of fitness trackers. The advantage of the MEMS-based sensor is a lower power requirement and lower sensitivity to artifacts, which can strongly influence photoplethysmography-based measurements, such as misplaced or ill-fitting sensors [134]. It is expected that MEMS-based heart rate monitoring will become a larger area of interest in the future with the advent of self-powered sensors [135,136].

**Figure 12:**
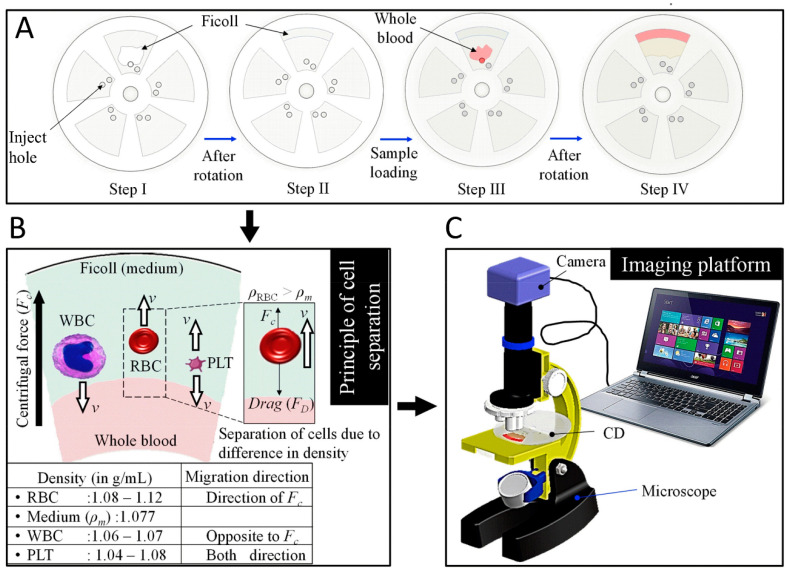
Representations of the experimental process of using spinning disk for blood component separation. (**A**) Spinning process including: 1. putting medium (Ficoll) into the disc sections. 2. spinning the medium. 3. putting whole blood sample into the disc sections. 4. spinning the blood sample. (**B**) Illustration of blood components distributions after the spinning. (**C**) Image captured for process via a computer. The achievement of automatic counting is assisted by the counting grid etched on the outer surface of the disc sections. Reprinted with permission from [138]. Copyright © 2019 Elsevier B.V.

Whilst technical advances in biosensors, including improvements in sensitivity and limit of detection, are important, large scale commercial viability is perhaps more important for functionality. For very expensive or technical biosensors, such wide scale implementation is virtually impossible, limiting the practical use of such technology. This is particularly an issue in developing countries, where expense and complexity can be prohibitive, leading to an excess of unnecessary deaths. Innovative solutions to such issues are being developed, notably by Prakash et al., who focus on low cost, simple solutions to challenging problems. Recently they created an innovative paper alternative to commercial centrifuges, separating red blood cells from plasma to provide a pure enough sample for malaria identification [137]. A similar approach has been taken to create an alternative to expensive automated haematology analysers [138]. Figure 12 demonstrates the operating principle of this device, as red blood cells have a higher density than blood plasma, they are pushed to the outside of the disk, white blood cells in contrast, have a lower density and move towards the centre of the disk. After separating blood into its components, automated counting can then by accomplished using Image J [138]. Another solution to a lack of equipment is to adapt equipment already available. This has been a focus for Prakash et al., capturing audio recordings of mosquitoes with the microphones of mobile phones and using the species-specific frequency of wingbeats to identify the species present [139]. This approach can generate a huge amount of data to help map the global distribution of mosquitoes.

## 3. Electrical Biosensors

### 3.1. Impedance Techniques

Impedance-based biological measurements are generally straightforward to implement, but significantly harder to interpret [140]. They involve the application of an alternating electric field and examining the impedance response of a single cell or group of cells to that field [140,141]. This can be used to track cell processes, monitor analyte concentrations or to follow monolayer growth and migration [141,142]. These systems have the advantage of a real-time response and can be used with an array of electrodes to achieve spatial resolution [143,144]. Furthermore this technique may be used in combination with microfluidics to achieve high throughput flow cytometry without the need for labelling [145]. There exist however some serious drawbacks to this method. It is not always easy to distinguish between different cell types, given the dependence on cell size and dielectric properties [140]. It can also be a challenge to examine single cells, given current technology constraints [146]. Nevertheless, it is clear that bioimpedance analysis has a strong potential for future biosensors.

The first investigations of biological impedance date back to the early 20th century, with Höber investigating the conductivity of erythrocytes at high and low frequencies [147]. This was followed by postulates developed by Berstein in his work of 1912 [148,149]. Before any understanding of cell structure, this work hypothesised the following: that living cells are composed of an electrolytic interior covered by a thin semi-permeable membrane, which creates an electrical potential across the membrane. This potential is reduced during activity by an increase in ion permeability of the membrane [150]. Since then, measurements of impedance, done over a range of different frequencies, have formed the basis of many closely related biological techniques. Among them, bioelectrical impedance analysis, which is a whole body measurement can determine the fat percentage of an individual, due to the insulating properties of body fat [151]. There remain, however, issues with this technique, mainly due to physiological variation from person to person [152,153].

Electric cell-substrate impedance spectroscopy (ECIS) is another important technique. ECIS is used to measure the impedance of cells adhered to an electrode due to an applied AC field, usually at multiple frequencies [140]. It is a widely used technique to monitor whole cell and cell monolayer impedance during culture [142,154]. The history of ECIS dates back to 1984 with Giaever et al. [155] using evaporated gold electrodes to probe cell movement over a surface electrically. This was expanded to develop an electrical model of mammalian cells [156] and further to enable identification of different protein coatings, and demonstrate a theoretical vertical resolution of 1 nm [157] It was further used to examine cell substrate interactions in a highly sensitive, real-time manner [158].

The advantage of ECIS as a system is its capacity for real-time monitoring of a cell culture, which is often a challenge, as many mammalian cells of interest are highly sensitive to their environment. Exposure to sub-optimal temperature, pH or other conditions can quickly cause cell death; and whilst gold electrodes are not the optimal surface for cell culture, the surface is inert and sufficiently biocompatible for cell survival if external conditions (temperature, CO_2_ level etc.) are maintained [159,160,161]. However, it is still not the ideal surface for cell culture [162], particularly in the longer term. Therefore, in recent years, attention has turned to softer, stretchable and flexible surfaces for cell impedance sensing. By using a stretchable surface, it becomes possible to examine the motion of the cell due to its effect on the underlying substrate. There have been a few different approaches to this: Dekker et al. have used silicon lithography techniques in combination with PDMS to create a modular stretchable device to simultaneously measure strain and monitor cells electrically [144,163]. The intelligent design of this system can allow various levels of strain to be applied to a cell culture whilst monitoring their response. A similar, though less controllable approach has been taken by Bernardeschi et al. who used a pre-stretched PDMS membrane to examine impedance changes under compression, presenting a new method for performing mechano-transduction in cells [164]. Other approaches to soft ECIS type measurements come from Kunduru et al. who have used an electrospun polystyrene platform coated with conductive polypyrrole and a C reactive protein antigen to demonstrate impressive sensitivity down to 1 pg mL^−1^ [165].

One of the reasons to monitor cell culture in real time is that, during the lifetime of a cell, it develops a localised potential as part of a variety of different processes. For example, when extending part of the cell membrane (in order to expand or move), the cell will accumulate calcium ions locally in that area in order to soften the actin filaments that make up the cytoskeletal structure near that point in the membrane. The membrane can then expand outwards to accommodate the local pressure, creating an outshoot arm-like structure. The reduction in cell potential via removal of the calcium ions reforms actin filaments and re-strengthens the membrane [166,167]. Thus the monitoring of this process enables an understanding of cell mechanisms of movement and growth. This process can be monitored electrically, due to the change in potential within the cell altering its impedance, as demonstrated by Wang et al. who synchronised cell cycles to track the changes in impedance during a cell’s development [168]. ECIS is also often used in the form of a wound healing assay, such as that demonstrated by Wang et al. who used self-assembled monolayers to control wound healing [169]. The migration process only takes a couple of hours, and so to get meaningful information, the process needs to be continuously monitored, which is where ECIS comes in useful.

**Figure 13:**
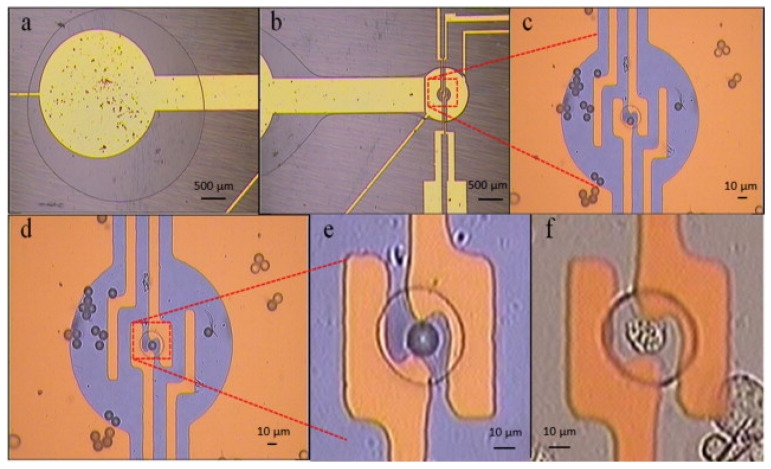
Device using dielectrophoretic transportation with cell trapping in cavity. (**a**) Electrode area where droplet is placed. (**b**) Cell is transported toward the cavity electrode. (**c**–**f**) Single cells are captured in cavity electrode for impedance measurement. Reprinted with permission from [170]. Copyright © 2013 Elsevier B.V.

Despite the advantages of ECIS, it does still have some drawbacks. The scale of the electrode systems and the random nature of cell deposition do not allow the probing of an individual cell. Therefore, in order to successfully examine a single cell, some sort of separation process must be applied to isolate a single cell. This has been successfully demonstrated by Chen et al. who used the technique of dielectrophoresis (discussed in Section 3.2) in order to separate an individual cell from a suspension and channel it into a carefully constructed cavity [170]. Figure 13 illustrates this process, following the movement of a single cell into the cavity. Once in the cavity the impedance of the cell could be measured over a range of frequencies and compared to computational models of the system. In a similar vein, Bhatt et al. used dielectrophoresis to concentrate DNA chains prior to impedance analysis [171]. A huge benefit to this technique is that the same electrodes can be used for the dielectrophoresis as the impedance analysis, so very little extra input is required.

**Figure 14:**
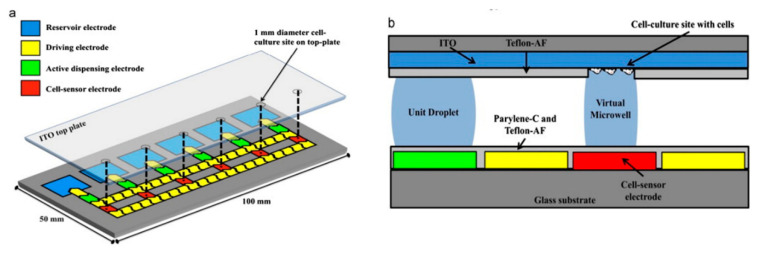
(**a**) Schematic view of a digital microfluidic device for cell culture and impedance measuring. The device contains 6 patterned cell-culture sites named virtual microwell and 66 electrodes. (**b**) Side view of the device. Reprinted with permission from [146]. Copyright © 2012 Elsevier B.V.

Other techniques to sort a cell population have been created using microfluidics. Shih et al. have used digital microfluidics to move and sort individual cell droplets to form “virtual microwells” whose population can then be measured both electrically and optically in real time [146]. The movement is accomplished by using an electric field to decrease the wetting angle of a droplet on a hydrophobic surface, causing the droplet to flow in the direction of the less hydrophobic surface, which is controlled by the electric field. Figure 14 demonstrates the construction of the system used by Shih et al. Such a system is highly interesting, and could be very useful for future diagnostics by separating out the population of interest [172]. More recently, true single cell separation has been achieved by Liu et al. who have effectively employed a microfluidic channel to sort a single cell suspension into individual wells [173]. If this were to be employed with electrodes embedded within the wells (as previously demonstrated [174]), it would permit more detailed information to be achieved with relative ease. The earlier work of Han et al. has demonstrated this type of technology is suitable for the analysis of cell lines, demonstrating sufficient sensitivity to distinguish successfully between early stage, invasive and metastasized human breast cancer cell lines [174]. This could prove to be a powerful tool for diagnostics, which is currently lacking in this area (with the relatively slow and inaccurate mammogram being the current default) [175].

A different approach to single cell analysis is to use a high throughput dynamic system rather than trapping a single cell. Whilst this does risk having a relatively high noise, it has been proven effective at identifying, counting and sizing red and white blood cells, with a simple and cost effective set up, that can detect over 400 cells s^−1^ [145]. Claudel et al. have demonstrated a similar system that is sufficiently accurate to determine both the size and cytoplasm conductivity of yeast cells on the order of 3 µm. This was achieved by using differential calculations at different frequencies to distinguish between media conductivity and cytoplasm conductivity [176]. A similar design was originally used by Gawad et al. to demonstrate particle sizing whilst achieving a throughput of 100 samples s^−1^ [177].

**Figure 15:**
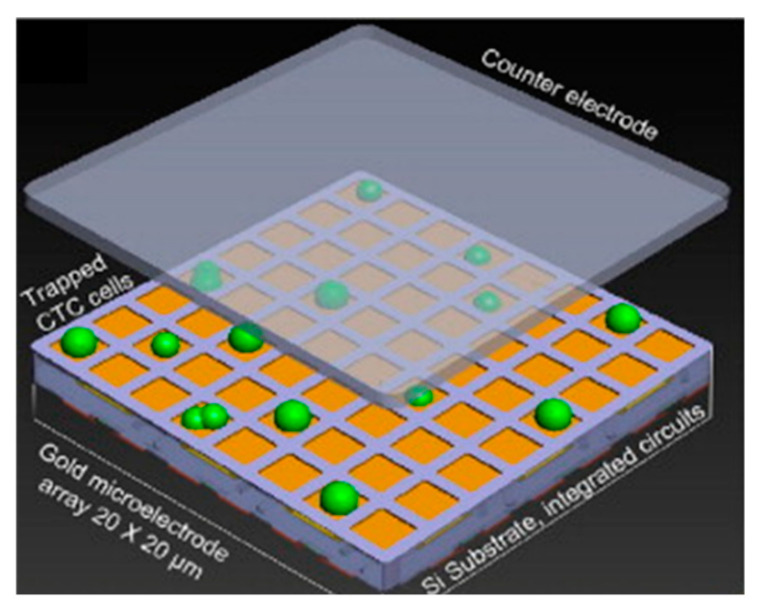
A schematic microelectrode array for cell counting. Reprinted with permission from [143]. Copyright © 2012 Elsevier B.V.

Impedance-based techniques have also been employed to map and count cell populations. Chen et al. demonstrated a CMOS chip with over 9000 gold electrodes for simultaneous measurement of impedance. The arrangement of the system is shown in Figure 15. with the working electrodes underneath the cells and the counter electrode on top. This construction enables spatial correlation, in essence forming a heat map [143,178]. The specificity of this system is currently unclear, but the impedance mapping was shown to have a fairly high degree of fidelity, making this a viable tool in future cell culture experiments. Its use has been further expanded by the long-term (3 month) culture of neuronal networks on a commercially available CMOS multielectrode array, allowing both detection and simulation of the resulting neuronal networks [179].

Recently, investigations have begun into biosensors to measure the response of cardiomyocytes to a variety of stimulants. This is important commercially, as a large proportion of pharmaceutical research becomes obsolete in the final stages when adverse effects on cardiac tissue become known. Platforms to easily measure this response would significantly increase efficiency. Kanade et al. have demonstrated a platform for simultaneous mechanical and electrical monitoring of cardiomyocytes, using a microcantilever combined with ECIS [180]. Multiple sensor platforms such as lab-on-a-chip (LOC) technologies are becoming increasingly prevalent with the advancement and integration of microfluidics and relatively low-cost and facile production methods such as aerosol-jet printing [181] and inkjet printing [182,183]. This has led to the production of low-cost, multifunctional printed microfluidic platforms with capabilities including cell separation, concentration, single particle trapping and impedance analysis [184].

### 3.2. Dielectrophoresis

Dielectrophoresis (DEP) is a technique that utilises an inhomogeneous electric field to move particles according to their polarisability [185,186]. It can be an effective, high-throughput method for manipulating different cell populations through differences in their polarisability [187,188]. It is a non-invasive technique, and may be used without detriment to the cells under examination [189]. Due to these desirable characteristics, DEP is increasingly being utilised as part of other sensors to manipulate and move single cells [170,190]. However, it can prove difficult to fully purify cell populations with DEP, due to inherent differences between cells and the unintentional effects that the electric field can have on the surrounding media [191]. Nevertheless DEP is starting to be used on its own to characterise cells, based on their crossover frequency or surface polarisability [190,192].

**Figure 16:**
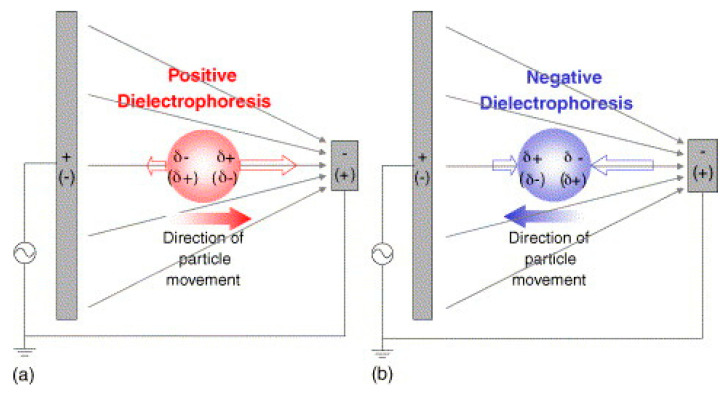
Directions of cell movement based on dielectrophoresis interactions. (**a**,**b**) show the responses of positive/negative dielectrophoresis. Reprinted with permission from [186]. Copyright © 2005 Elsevier B.V.

DEP refers to the force on a dielectric particle in response to an applied non-uniform electric field [193]. To be dielectric, a particle needs to be both insulating and to have a high relative permittivity [187]. Any particle satisfying both these conditions will experience a dielectrophoretic force. The force acts as follows: both ends of the particle develop a polarisation, but due to the non-uniform field, one end will be larger than the other; the size and sign of the polarisation at each end is determined by the polarisability of the particle compared to the external medium [194]. If the particle is more polarisable than the medium then the particle will experience positive dielectrophoresis and move towards the higher intensity field. If the particle is less polarisable then it will move towards the low intensity field (as the surrounding medium in effect moves towards the higher intensity field) [186,195]. This is illustrated in Figure 16.

The history of DEP dates back to the 1950s with Pohl’s examination of the movement of suspensions in divergent electric fields [196]. This continued with Pohl using DEP to precipitate, stir, pump and separate suspensions [197]. This work further progressed and in 1966, Pohl demonstrated the use of DEP to separate live and dead yeast cells [193], the first such demonstration of DEP being used in biotechnology [198]. Progress in the field was slow until the 1990s owing to the limitations in fabrication techniques limiting the miniaturisation of the electronics used, therefore requiring high electric fields to produce meaningful motion [198]. The advent of microfabrication techniques generated renewed interest in the technique [199,200,201]. This led to the literature becoming dominated by publications based on planar metal electrodes, and since 2008 interest has plateaued at around 300 publications a year [198,202]. More recent research in the area has focused on the separation of proteins and DNA rather than the cell separation that marked the early work [202,203,204]. The success of DEP is sufficient that there are now commercially available systems to sort and isolate cell populations based on DEP [205].

DEP may be carried out using insulating materials instead of electrodes, referred to as insulating DEP. There are some advantages to this including improved ease of fabrication (as it is possible to construct the channel out of a single material), reduced fouling of the test region, lack of electrochemical processes on the electrodes and the resultant capacity to operate at lower frequencies than electroded DEP [201,206,207]. However, the separation of the electrodes from the region of interest means an increased voltage is required to generate DEP, which often results in cell death [185,207]. The higher electric field for insulating DEP also results in unintended effects including Joule heating, alternating current (AC) electro-osmosis and AC electrothermal hydrodynamics. All of these can result in fluid motion that interferes with the intended process [201,208]. The insulator material can also make it challenging to predict the shape of the electric field [207,209] which may make modelling a challenge, so whilst insulating DEP has some benefits, its drawbacks make it currently less suitable than electroded DEP.

**Figure 17:**
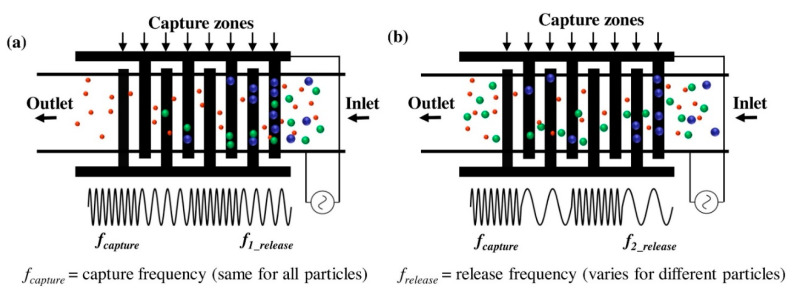
Illustration of the working mechanism for a frequency hopping based dielectrophoresis cell filter over interdigitated electrodes, proposed by Modarres. A higher applied frequency traps all sized particles trying to pass over the electrodes while various lower frequency (**a**)/(**b**) release different sized particles to achieve size filtering. Reprinted with permission from [211]. Copyright © 2019 Elsevier B.V.

Improvements to the fabrication process for electroded DEP are increasing its advantages over insulating DEP. One approach is to use soft lithography in place of silicon lithography. This has been demonstrated with high efficiency by Nie et al., using PDMS and silver mix to form the structure, enabling separation of 99.9% of impurities with a high flow rate of 260,000 cells per minute [210]. Another option is to take advantage of a property of DEP; that it does not depend upon the sign of the applied field, only the relative polarisabilities. This means that DEP can be used with an alternating current (AC). This is important for the application of DEP to cells, as the capacitive effects of the cell membrane are negligible at higher frequencies [140], enabling separation of cell populations based not on the external media, but on the size of the cell and conductivity of the cell cytoplasm [188]. This has been utilised by Modarres et al. in a “frequency hopping” DEP to capture cells at one frequency and selectively release the unwanted cells at another [211], as shown in Figure 17. This design lead to a capture efficiency of over 80% of the target cells. The work of Zhao et al., also used an AC field to characterise the difference in DEP force experienced by both live and dead yeast cells at different frequencies [188]. This may in the future be used to characterise cells by their crossover frequency (the frequency at which the DEP force changes from positive to negative). Current work on characterising cells using DEP includes an assessment of surface polarisability by Wang et al. which may be used to assess phenotype [192].

### 3.3. Field Effect Transistors

**Figure 18:**
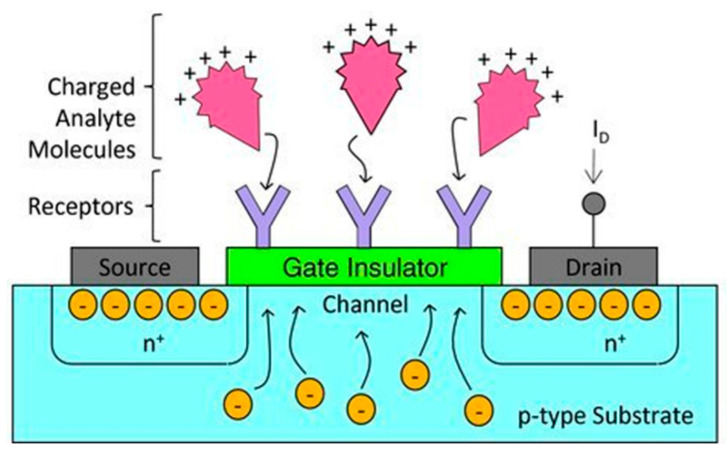
The operation mechanism of an FET biosensor. Reprinted with permission from [214]. Copyright© 2011 WILEY-VCH Verlag GmbH & Co. KGaA, Weinheim.

The applications of field effect transistors (FETs) extend beyond the electronics industry into the realm of high-sensitivity biosensors [212,213]. They operate based on a semiconductor between a source and drain terminal, whose impedance is changed via the field effect of an applied electric field (via a gate terminal) [214]. When a molecule binds to the surface receptor, it changes the surface potential, with the corresponding change in channel width altering the current between source and drain, as shown in Figure 18 [214]. In recent years, miniaturisation of FETs (mainly through processes developed for the electronics industry) has greatly reduced the limit of detection of these devices [212,213]. The reduction in the size of the channel and the distance between the binding event and the channel has largely contributed to this improved sensitivity. Miniaturisation has also led to increased applications of these sensors as arrays, to detect multiple analytes, or in applications demanding a small scale [215]. One of the drawbacks of FETs is that whilst suitable for short term in vitro application, they are sensitive to environmental change, and not always biocompatible, so have not yet been made functional for long term in vitro use [216,217].

The first use of FETs in biosensors came in 1972 when Bergveld developed the ion-sensitive FET (ISFET); a device which utilised an aqueous solution between the gate electrode and the device body to determine ion concentrations by their electrical double layer formation [218,219,220]. Simultaneously, Matsuo worked on a similar device with a slightly different design [221]. Through the 1970s, ISFETs were further verified mainly in use detecting the pH of various solutions [222]. Much of this interest was generated with the aim of using ISFETs as physiological sensors, with some success [223,224]; although the range of physiological processes that could be observed at that time was limited to those that produced a pH change.

Over time the use of ISFETs was expanded to include other ions, for example heavy metal ions such as cadmium [225]. One of the main advantages of ISFETs is that they are non-selective, and may be used with any suitable ionic solution [226]. Therefore, if processed with a suitable selective membrane (e.g., by using an ionophore) they are able to detect multiple different ions in solution [227]. One drawback to this is that with the sensor being sensitive to multiple different types of ions, any leakage of solution or change in the external environment affecting the pH of the solution, will change the ISFET reading. Over the longer term, this is particularly an issue, as hydrogen ions are difficult to prevent from permeating a membrane due to their small size [227]. Fortunately, an advantage for ISFETs (and other FET-based sensors) is that due to the huge commercial interest in transistor technology, miniaturisation of FETs is now very straightforward. This enables many more ISFETs to be placed on the same device, forming multi-sensor arrays [215]. Furthermore the work of Estrela et al. has illustrated the suitability of this technology for low cost, miniature DNA sensors [215].

**Figure 19:**
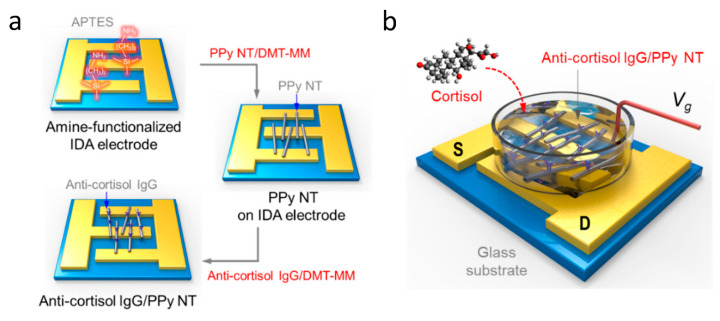
(**a**) Schematics of the fabrication procedure to integrate immunoglobulin G (IgG)/polypyrrole nanotube FET-type biosensor. (**b**) S and D represent source and drain electrodes, while the gate electrode was immersed in the buffer as a liquid-ion gate. Reprinted from [233] under CC BY 4.0 (https://creativecommons.org/licenses/by/4.0/).

As the use of ISFETs expanded, so did the variety of different analytes being studied. This led to the development of the first enzyme FETs, initially using a penicillinase enzyme bound over an FET in order to detect the presence of penicillin [228,229]. Since then a wide range of enzymes and analytes have been tested, including cyanide ions [230], lactic acid [231] and adenosine triphosphate [232], demonstrating great versatility. The functionalisation of FET surface is what has enabled improved analyte detection and specificity. With improving technology, attention has turned back to the medical applications of FETs; with multiple different receptors able to detect a variety of proteins and small molecules typically found in blood [214]. Furthermore, FET surfaces can be functionalised effectively through the formation of nanowires for the channel between source and drain. This has been proven to decrease the limit of detection down to incredibly small concentrations by Kim et al. with the use of polypyrole nanotubes [233]. The nanotubes were deposited across interdigitated source and drain electrodes and functionalised with immunoglobulin G. When the cortisol analyte was added, it bound to the immunoglobulin G and increased the current between source and drain, as illustrated in Figure 19. More common, however, is the use of silicon nanowire FETs in biosensors [234,235]. The nanoscale functionalisation of FETs has greatly increased interest, leading to reported limits of detection down to 10 fM for dengue virus PNA [213] and even 1 fM for human micro RNA [236]. Whilst not all studies have not shown as small limits of detection, there have been some studies showing similar ranges of detection, with Presnova et al. using gold nanoparticles to functionalise the surface of a silicon FET demonstrating a limit of detection of 0.7 fM for prostate specific antigen [212].

## 4. Conclusions

In this review we have examined some of the exciting technologies and developments in the fields of electrical and mechanical biosensors. Some of the highlights of these fields are shown in Table 1, which compares the detection limit, analyte, bioprobe and some of the advantages and disadvantages of the different biosensors covered here against other leading biosensors. As shown in Table 1, electrical and mechanical biosensors have certain important advantages when compared to optical based biosensors, in that they generally provide fast and real-time measurements, enabling rapid assessment of an analyte of interest. The responsiveness of many electrical and mechanical biosensors is largely due to two factors: integrated circuits (usually fabricated in gold or into silicon chips), and reductions in size, reducing the quantity of analyte required to induce a meaningful change (as analyte can only bind to the surface, and surface-to-volume ratio increases with increasingly small sizes). These two improvements are driving an incredible improvement in biosensor sensitivity. Micro and nano scale sensors are able to detect compounds at unprecedentedly small concentrations, down to the region of fg mL^−1^. In fact, one suspended microchannel resonator has been shown to detect the binding of a single 20 nm gold nanoparticle [53], however the throughput of this sensor is too slow to be practical.

There are some important differences between electrical and mechanical biosensors. Mechanical biosensors are generally more complex to fabricate and model, as they require a transducer of some kind, whereas electrical biosensors produce their own signal. Mechanical biosensors are more widespread, QCM and SAW based biosensors [237] in particular are important commercial scientific tools; but photoacoustics [238] and microcantilevers [239] are also becoming more common, whereas in electrical systems only ECIS has a significant commercial market [240]. Electrical biosensors are more likely to provide general information about an analyte (polarisability, impedance etc.) than mechanical biosensors which as a rule provide information about the concentration of analyte. In a similar vein, due to the required functionalisation, mechanical biosensors are more likely to be specific to a given analyte.

In the future, it seems likely that the development of microfluidics will have a significant impact on biosensing technology. Currently it appears that electrical biosensors are better placed to take advantage of this development, because it has already been demonstrated that FETs, ECIS and DEP can be successfully integrated into microfluidic devices [146,170,235]. However, for mechanical biosensors, photoacoustics is the only field that is well positioned to take advantage of new microfluidic technology [70].

## 5. Outlook

The question therefore is, what are the goals of improving detection limits? The long-term aim is geared towards improving diagnosis for diseases, such as prostate cancer (hence the prevalence of biosensors aimed at prostate specific antigen) which has a very small threshold for concentrations considered abnormal (~4 ng mL^−1^). However, this threshold has already been passed, only six of the sensors presented above do not reach this threshold, and all of the sensors specific to prostate specific antigen do. The focus, therefore, should now shift to practically implementing these sensors, to improve testing procedures and diagnoses. This is where the most important differences between biosensors emerges. Those with prohibitively high cost, complex equipment or fabrication and requiring large amounts of training are unlikely to see a wide uptake. Sensors fabricated using silicon fabrication technologies have an obvious advantage over others, due to the improvements in these techniques by the electronics industry, making silicon-based sensors cheap, reliable and easily integrated. This is particularly the case for MC and FET technology, but is also possible for impedance-based sensing, particularly when combined with dielectrophoresis to concentrate the analyte [241]. 

**Table 1:** Summary and comparison of electrical and mechanical biosensors against other leading biosensor techniques.

Field	Ref.	Detection Limit/fg mL^−1^	Analyte	Bioprobe	Analytical Surface	Notes	Advantages	Disadvantages
Microcantilever	[30]	50	Prostate Specific Antigen	Antibody	Silicon	Trampoline shaped resonator, vacuum required	High sensitivity, label free, commercially available, easy fabrication.	Complex preparation, use of lasers and preference for vacuum conditions.
	[31]	4	Estradiol hormone	Antibody	Silicon	Array of microcantilevers, optical lever type detection		
	[53]	21,000	Gold Nanoparticle	-	Silicon	Suspended nanochannel resonator, low flow rate (1 pL s^−1^)		
Photoacoustic	[58]	775,000,000	ONOO^-^ marked with CyBA	Small molecule	In vivo	Commercial LED Photoacoustic imaging system at 1 cm depth	Non-invasive imaging and detection, real-time measurements, useful for flow cytometry.	Lasers commonly used, bulky, expensive.
	[57]	0.9	HF	-	Silicon	Microcantilever transducer		
	[70]	150,000	Malaria infected RBC	-	In vivo	Photoacoustic flow cytometry		
Micropillar	[80]	-	HeLa	Fibronectin	Silicon	Gold disk coated silicon pillars, traction force 1 nN LoD	Effective force sensors, could use cells as proxy, could be developed with electrical measurement.	Currently complex image processing, sensitivity needs improvement, not physiological conditions.
	[242]	-	Mouse embryonic fibroblasts	Fibronectin	PDMS	Silicon templated PDMS pillars, traction force 0.1 nN LoD		
	[99]	-	Water flow	-	Silicon on PZT	Si array embedded in PDMS, water velocity LoD 8.2 µm s^−1^		
QCM	[123]	130,000	C-reactive protein	Antibody	Gold on Quartz	Indirect competitive reaction	Commercially available, cheap, real time measurements, label free.	Sensitivity limited by size constraints; surface functionalisation remains key issue.
	[121]	50,000,000	DNA	DNA	Gold on Quartz	Complementary DNA immobilised with sulphur on gold		
	[243]	14.3	Lysozyme	DNA	Gold on Quartz	Biocatalytic precipitation amplified		
SAW	[126]	3,500,000	Bacterial endotoxin	DNA	Graphene on Quartz	Single layer graphene	Commercially available, real-time measurements, potential for higher sensitivity than QCM, label free.	Surface functionalisation still issue, relatively long preparation.
	[108]	310,000	Carcinoembryonic antigen	Antibody	Gold on Quartz	Chemically modified gold, stable over 30 days		
	[114]	100,000,000	E. Coli	Antibody	AlN	Flexible AlN on PEN, for polymer RFID food packaging		
ECIS	[165]	1000	C-reactive protein	Antibody	Polypyrrole on PS	Conductive coated polystyrene electrospun mat, low cost	Spatial resolution possible, real time response, label free, simple, cheap.	Challenge processing and interpreting data, difficult to measure single cells.
	[244]	3,300,000	Okadaic acid	HeLa Cells	HeLa cells on Gold	Cells used as proxy for toxin		
	[245]	200	E. Coli	Antibody	Gold	Functionalised with self-assembled monolayer template		
Dielectrophoresis	[246]	1000	Cardiac troponin I	Antibody	Carbon nanotube	Dielectrophoretic enhancement, impedance measurement	Can purify molecules of interest, non-invasive, commercially available, easy fabrication.	By itself not sensitive, can cause cell death, affected by environmental factors.
	[241]	3.4	Prostate Specific Antigen	Antibody	Silicon nanowire	Dielectrophoretic enhancement, impedance measurement		
	[247]	27,000,000	Trypanosome	-	Gold on Glass	Spiral electrodes concentrates analyte, manual visual count		
FET	[212]	23	Prostate Specific Antigen	Antibody	Silicon nanowire	Surface modified with Gold nanoparticles	Extremely sensitive, commercial technology, real time measurements, simple interpretation.	Sensitive to environment.
	[213]	1	Micro RNA	DNA	Silicon nanowire	PNA functionalised surface		
	[248]	3.2	DNA	DNA	Carbon nanotube	Single strand DNA functionalised surface		
SPR	[23]	10,000	Cardiac troponin T	Antibody	Gold	Modified gold with carboxymethyldextran hydrogel	Wide range of analytes, small sample volumes.	Dependent on surface functionalisation, requires knowledge of reaction mechanism, slow.
	[249]	1,500,000	C-reactive protein	E. Coli	Gold	Autodisplaying E. Coli as proxy		
	[250]	68,000	Cardiac troponin I	Antibody	Gold	Chemically modified gold		
Electrochemical	[251]	72,000,000	Glucose	-	ZnO nanorods	CuO nanoparticle modified	Simple interpretation, commercially available, well characterised.	Increasingly small gains, complex fabrication required for high sensitivity.
	[252]	55	DNA	DNA	Gold nanorods	On Graphene Oxide base		
	[253]	5,400,000	Glucose	-	Nanocomposite	Graphene, Ni and polyvinyl pyroldine nanocomposite		

As conventional electrochemical biosensors rely on a metal electrode surface, and SPR on gold coated glass, neither of these techniques is well suited to taking advantage of such manufacturing techniques. One of the other major developments in biosensor technology, is the range of methods developed for functionalising a sensor surface. This is important as the surface binding of the analyte in question determines the specificity of the sensor. It is no surprise therefore that DNA biosensors have been shown to have very low limit of detection, as the binding between two complementary DNA strands is both specific and very strong. Unfortunately, few antigen-antibody interactions are as specific or strong as complementary DNA binding, hence the interest in new surface functionalisation. One issue with this however is that increased specificity must be achieved for every target molecule of interest. In the future therefore, it is expected that biosensors with a more general applicability will be of interest, these include: Photoacoustic imaging, where signals can be generated from molecules of interest; impedance-based techniques, which has shown that a cell monolayer can be used as a proxy biosensor [128]; and dielectrophoresis, where the crossover frequency between a positive and negative DEP force could be used to characterise cells [188].

The final note of interest is the number of papers in recent years that overlap between two different sensors. This includes the use of: dielectrophoresis and ECIS [170], microcantilevers and ECIS [180], QCM and ECIS [125], microcantilevers and photoacoustics [58]. The benefit of these systems being that it is easier to correctly distinguish two separate species with two sets of tests to compare. It is expected that future work may produce more multi-sensor platforms, particularly in those where naturally overlapping fabrication routes exist, such as silicon fabrication processes. In particular, this paves the way for combining novel biosensors based on mechanical and electrical detections techniques with those based on optical and electrochemical mechanisms, for improved sensitivity, detection capabilities, and deployability.
